# The vitamin D status in a Chinese osteogenesis imperfecta population and its correlation with bone metabolic markers and bone density

**DOI:** 10.3389/fnut.2024.1390668

**Published:** 2024-08-05

**Authors:** Yunyi Jiang, Yazhao Mei, Yuan Tian, Li Shen, Shuqin Xu, Hao Zhang, Zhenlin Zhang

**Affiliations:** ^1^Department of Osteoporosis and Bone Disease, Shanghai Clinical Research Center of Bone Disease, Sixth People’s Hospital Affiliated to Shanghai Jiao Tong University School of Medicine, Shanghai, China; ^2^Clinical Research Center, Sixth People’s Hospital Affiliated to Shanghai Jiao Tong University School of Medicine, Shanghai, China

**Keywords:** osteogenesis imperfecta, vitamin D, bone mineral density, bone metabolic markers, China

## Abstract

**Objective:**

Studies on the baseline vitamin D levels in osteogenesis imperfecta (OI) patients before medication are scarce. This study assessed the vitamin D status of a population with OI at both the overall level and within different age groups. It correlated baseline 25-hydroxyvitamin D (25(OH)D) levels with other bone-related factors, biochemical markers, and bone density.

**Patients and methods:**

We collected 25(OH)D levels from 95 OI patients in East China (59 under 18 years old and 36 over 18 years old). Postmenopausal women and men over 50 years old are excluded. Measurements included body indicators, biochemical markers, and bone mineral density (BMD) assessed by Dual-energy X-ray absorptiometry (DXA). Data analysis was performed using SPSS 26.0.

**Results:**

In the overall population, among those under 18 years old, and among those over 18 years old, 87.4, 83.1, and 94.4%, respectively, were vitamin D deficient (<30 ng/mL), while 47.4, 40.7, and 58.3% had vitamin D deficiency (<20 ng/mL), respectively. In the overall population and among those under 18 years old, serum 25(OH)D levels were negatively correlated with age and parathyroid hormone (PTH) levels, and 25(OH)D levels (<10 ng/mL, 10–20 ng/mL, 20–30 ng/mL, >30 ng/mL) showed a negative correlation with BMI. In OI patients under 18 years old, serum 25(OH)D was negatively correlated with serum β-CTX levels. In adult male OI population, 25(OH)D levels were negatively correlated with OI severity (Type I, IV, III). No statistically significant correlation was found between 25(OH)D levels and BMD Z-scores.

**Conclusion:**

This study on OI in East China reveals significant vitamin D insufficiency and deficiency in baseline levels among pediatric, adolescent and adult OI patients. It assesses the correlation of 25(OH)D levels with various influencing factors, providing crucial insights into understanding the impact of OI on vitamin D status across different age groups and aiding in better clinical management of OI patients.

## Introduction

Osteogenesis Imperfecta (OI), commonly known as brittle bone disease, is a group of hereditary bone disorders characterized by low bone mass and bone fragility, leading to long bone fractures and vertebral compressions, accompanied by skeletal deformities and growth defects. Some patients may also exhibit extra-skeletal phenotypes, including blue sclera, conductive or sensorineural hearing loss, dentinogenesis imperfecta, pulmonary function impairment, cardiac valve abnormalities, and lax skin and ligaments (1).

Currently, the available treatment options for OI include fracture prevention, symptom control, and increasing bone mass. Bisphosphonates (BPs), the most commonly used medication for treating OI (2), have been proven to increase bone mass in OI patients and, to a certain extent, reduce the risk of fractures (3). They are the first choice for drug treatment in pediatric OI patients, moderate to severe OI patients, as well as mild OI patients with a history of recurrent fractures, low trauma fractures, or vertebral compression fractures, or severe bone pain (4). Regardless of the type of medication used, adequate vitamin D levels and proper calcium intake are essential; without these, any treatment is destined to be ineffective (5).

Vitamin D plays a crucial role in maintaining the balance of calcium and phosphate metabolism, as well as in the development and maintenance of bones, through its most biologically active metabolite-1,25(OH)_2_D (6). Bone absorption, modeling, and remodeling must be considered as vitamin D-dependent processes (7). Studies have shown that a deficiency in vitamin D is directly related to an increased risk and severity of fractures in healthy children (8). Providing supplementation to children identified as vitamin D deficient may have clinically beneficial effects on peak bone mass (9). Adequate intake of calcium and vitamin D may optimize the response to bisphosphonate treatment by preventing symptomatic hypocalcemia and enhancing the availability of the matrix for bone mineral accumulation (10). Ensuring adequate serum levels of vitamin D is often mentioned as a target in the supportive medical management of OI (11).

Serum 25-hydroxyvitamin D (25(OH)D), due to its high concentration in circulation and long half-life, is the primary circulating form of vitamin D and serves as the best clinical indicator of vitamin D status (12). It reflects the supply and reserve of both endogenous and exogenous sources of vitamin D (13). 25(OH)D, as a precursor of 1,25(OH)_2_D, indirectly affects calcium and phosphorus metabolism, PTH secretion, maintains bone health, and balances the levels of bone metabolism markers. Previous studies have explored the relationship between 25(OH)D and bone metabolism indicators, with studies indicating that lower levels of 25(OH)D are often associated with higher levels of CTX, indicating increased bone resorption (14). Low levels of 25(OH)D are often associated with higher levels of ALP, suggesting increased bone formation to compensate for mineral deficiencies (15). Studies showed that serum 25(OH)D levels below 30 nmol/L are associated with impaired calcium absorption across all age groups, increased risk of rickets in children, reduced bone mineral content (BMC), increased risk of osteomalacia in adults, and higher fracture risk in the elderly (10). Currently, based on the opinions of most authoritative academic institutions and experts worldwide (16), vitamin D status is classified into the following levels:

Vitamin D deficiency: 25(OH)D below 20 ng/mL (50 nmol/L);
Vitamin D insufficiency: 25(OH)D:21–29 ng/mL (52.5–72.5 nmol/L);
Vitamin D sufficiency: 25(OH)D above 30 ng/mL (75 nmol/L).

In recent years, scholars have hypothesized that populations with potential bone fragility should be particularly vulnerable to the additional challenges of low vitamin D status. Low levels of vitamin D might lead to decreased bone mass or exacerbate primary bone diseases. In 2008, Bowden et al. (17) conducted a study involving 85 children with osteopenia or osteoporosis (osteopenia was defined as lumbar vertebral BMD Z-score between −1.0 and −2.0 and osteoporosis as lumbar vertebral BMD Z-score less than −2.0) and found that 80% of them had 25(OH)D levels <30 ng/mL, and 21.1% had levels <20 ng/mL. Besides a negative correlation with PTH levels, no direct correlation was found between serum 25(OH)D levels and bone density, fracture rate, or other bone metabolism markers. Subsequently, a North American study by Edouard et al. (18) on 71 OI children showed a 52% prevalence of vitamin D deficiency. This study indicated that 25(OH)D levels ranging from 13 to 103 nmol/L were not associated with tissue morphometric parameters of bone mineralization or bone density. However, their subsequent study on 282 OI children indicated a 27% deficiency in 25(OH)D, and regression analysis revealed that for every 1 nmol/L increase in 25(OH)D levels, the LS aBMD Z-score increased 0.008 (18). A retrospective study by Wilsford et al. (19) on 80 OI children showed that nearly 80% had insufficient or deficient vitamin D levels. In southern Brazil, a study on 52 OI children and adolescents found that 35.5% had vitamin D deficiency and 51.9% had vitamin D insufficiency; 88.4% of cases were insufficient or deficient in vitamin D, and a correlation was observed between serum 25(OH)D concentrations and whole-body BMD and Z-scores (20). A study in southern Iran showed that 43.4% (10/23) of OI children had vitamin D deficiency, and there was no correlation between vitamin D levels and bone density parameters (21).

In studies involving adult OI patients and their vitamin D levels, Wekre and colleagues (22) found in their research on OI patients over 25 years of age that 17.6% (16/91) of the patients had 25(OH)D levels <20 ng/mL, with Type III OI patients showing significantly lower 25(OH)D levels than those with Type I and IV OI. In a cross-sectional study by Chagas et al. (23), 73% (19/26) of OI patients had 25(OH)D levels <20 ng/mL, while 8% (1/13) of Type III OII patients were considered vitamin D deficient, though there was no significant difference compared to the control group.

Currently, research on vitamin D levels in OI patients is mostly focused on children and adolescents under the age of 18, with relatively fewer studies on adult OI patients. Moreover, a significant portion of these studies’ subjects include patients who have previously used bisphosphonates or undergone vitamin D and multivitamin treatments, introducing more confounding factors related to 25(OH)D. Therefore, research on baseline data of untreated OI patients and the true prevalence of vitamin D deficiency across all age groups becomes particularly necessary. As indicated in the studies mentioned above, OI is a rare disease with significant heterogeneity in severity and subtypes, and the factors affecting vitamin D levels are numerous and complex. This leads to a high degree of variability in the prevalence of vitamin D deficiency among OI patients and in the correlation between vitamin D levels and influencing factors. The consistency of this correlation with the same influencing factors varies across different studies. Thus, further research on vitamin D levels in OI populations from different regions is extremely important. At present, there are no large-scale studies in China on vitamin D deficiency and its correlating factors in OI populations. This project has collected 25(OH)D levels from 95 OI patients (59 under 18 and 36 over 18) in East China, assessing vitamin D status both overall and across different age groups, and correlating it with other factors related to bone health, biochemical metabolic indicators, and bone density.

## Patients and methods

### Patients

This retrospective study included OI patients treated at the Sixth People’s Hospital of Shanghai. Inclusion criteria were a diagnosis of OI Types I, III, or IV; male patients under 50 years old, premenopausal women [in order to avoid the significant effects of estrogen and aging on bone density, as well as to exclude the significant reduction in skin vitamin D conversion after exposure to light in the elderly population (24)], and availability of initial serum 25(OH)D levels. Patients previously treated with bisphosphonates were excluded due to its significant impact on bone metabolism and density (25). The study involved 95 patients (59 under 18 years old, 36 over 18 years old), aged 2–45. For 92 patients (96.8%), serum 25(OH)D levels were collected alongside BMD measurements of the lumbar spine, total hip, and femoral neck. Sequence analysis of COL1A1 and COL1A2 were performed in 84 patients, revealing pathogenic mutations in 64 (81%). In 20 patients with negative gene analysis and 11 without DNA analysis, OI diagnosis was clinically confirmed based on frequent fractures, low bone mass, and features like blue sclera or dentinogenesis imperfecta. The study was approved by The Ethics Committee of Shanghai Jiao Tong University Affiliated Sixth People’s Hospital [Approval and 2022-KY-169(K)] and conducted with informed consent from legal guardians of children younger than 18.

### Biochemical measurements

Following the manufacturer’s protocol and specialized laboratory quality control procedures, fasting blood samples were collected between 8:00 and 10:00 in the morning and stored at −80°C. The following markers of bone metabolism were measured: serum calcium (Ca), phosphorus (P), alkaline phosphatase (ALP), intact PTH, 25(OH)D, procollagen type I N-terminal propeptide (P1NP), and the cross-linked C-terminal telopeptide of type I collagen β-CTX. Ca, P, and ALP were measured using the Hitachi 7,600–020 automatic biochemical analyzer; other compounds were measured using the following kits (all from Roche Diagnostics): the intact PTH kit for PTH, the 25-hydroxyvitamin D3 kit for 25(OH)D, the total P1NP kit for P1NP, and the -Cros-Laps kit for β-CTX. Serum osteocalcin (OC) was measured by Biomedica Laboratory (Biomedical Medizinprodukte GmbH and Co-KG) using a sandwich-type ELISA.

### Anthropometric measurements

Height was measured using a wall-mounted stadiometer, accurate to the nearest 0.1 cm. For infants and children unable to stand, length in the supine position was used. Weight was measured using digital scales for infants and mechanical scales for older children and adults (Healthometer, Bridgeview, IL). The measurements were then converted into age and gender-specific Z-scores, aligned with the standards for Chinese children and adolescents aged 0 to 18 years (26).

### Dual-energy X-ray absorptiometry

Dual-energy X-ray absorptiometry (DXA) was used to measure the BMD (g/cm^2^) of the lumbar spine (L1-L4), left femoral neck, and total hip. All subjects were assessed using the Lunar Prodigy device (GE Lunar Corp). The Lunar device was calibrated daily. Throughout the study, all DXA scans were performed by the same trained technician. The results were then converted into age and gender-specific Z-scores (27, 28). This methodology enables an accurate evaluation of bone health across different age groups and genders.

### Statistical analysis

Continuous variables were tested for normal distribution using the Kolmogorov–Smirnov test. Normally distributed data (BMD Z-scores) were presented as mean ± standard deviation (SD), and differences between groups were compared using student-t tests and one-way ANOVA. Non-normally distributed data were represented as median (range) and compared between groups using the Mann–Whitney U test. Spearman correlation detection is used for correlation analysis between non normal data, and Kendall’s correlation analysis is used for correlation analysis between grade data to examine the relationship between 25(OH)D levels and other factors. Statistical analyses were performed using SPSS software (version 26.0; SPSS Inc., Chicago, IL, United States). A two-tailed *p*-value of <0.05 was considered statistically significant.

## Results

**Figure 1:**
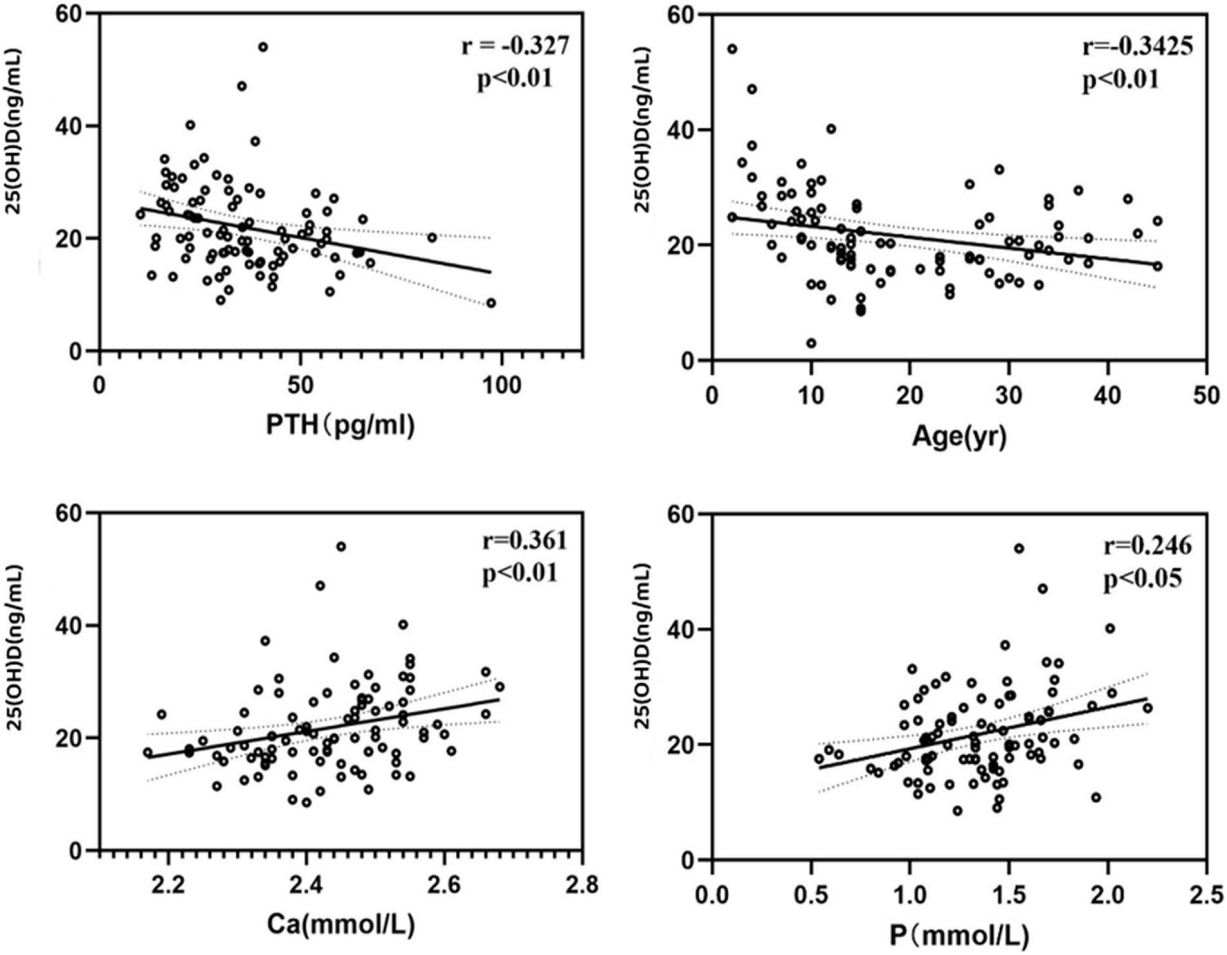
The correlation between overall level of 25OHD and other factors.

In our study, overall (n = 95, male 63/female 32), the average serum 25(OH)D level was 21.66 (±7.93) ng/mL (Table 1), ranging from 3 to 54.03 ng/mL. 83 patients (87.4%) had serum 25(OH)D concentrations below 30 ng/mL, 45 patients (47.4%) had levels below 20 ng/mL, indicating vitamin D deficiency, and 3 patients (3.2%) had levels below 10 ng/mL. Serum 25(OH)D levels were negatively correlated with age (r = −0.3425, *p* < 0.01) and PTH levels (r = −0.327, *p* < 0.01), and positively correlated with serum calcium (r = 0.361, *p* < 0.01) and phosphorus levels (r = 0.246, *p* < 0.05) (Figure 1). The serum 25(OH)D levels were categorized into four levels: severe deficiency (<10 ng/mL), deficiency (10–20 ng/mL), insufficiency (20–30 ng/mL), and sufficiency (>30 ng/mL). A negative correlation was observed between BMI and 25(OH)D level categories (r = −0.167, *p* < 0.05). Overall, there was no correlation between serum 25(OH)D levels and seasonality (Considering clinical observations and the sunshine and climate in eastern China, the seasons are divided into two groups: spring: March to May/summer: June to August and autumn: September to November/winter: December to February), OI severity classification (Type I 66/84, Type IV 12/86, Type III 6/84), or COL1A1/COL1A2 gene mutation type (nonsense 11/64, missense 24/64, frameshift 18/64, INTRON mutation 11/64).

**Table 1:** General characteristics and biochemical parameters.

Variables	*n*	All	*n*	≤18	*n*	>18		*p*
						Male	Female	
Sex (male/female)	95	63/32	59	43/16	36	20	16	<0.05^b^
Age (yr)			59	11 (8,14)	20/16	30.8 ± 6.5	31.8 ± 6.3	0.208
Height(cm)						165.47 ± 8.31	152.56 ± 7.11	<0.05^b^
Height(z-score)			59	−0.44(−1.51,0.2)				0.945
Weight(kg)					20/16	65 ± 11.46	49.78 ± 7.22	<0.05^b^
Weight(z-score)			59	0(−1.48,0.19)				0.871
aBMD L1-L4	75	−0.67(−1.94, −0.32)	51	−0.71(−1.93,0.5)	12/11	−1.49(−2.21,0.23)	−0.93 ± 1.2	>0.05
(z-score)								
aBMD femoral neck (z-score)	76	−0.69(−1.86,−1.0)	52	−1.08(−2.39,0)	13/12	−0.52 ± 1.69	−0.67(−0.96,−0.41)	>0.05
aBMD total hip (z-score)	76	−1.01(−1.81,−0.21)	51	−1.41(−2.26,−0.18)	13/12	−0.74(−1.67,0.16)	−0.89 ± 0.68	>0.05
Serum 25(OH)D (ng/mL)	95	20.19 (16.86,26.38)	59	22.71 ± 9.0	20/16	22 ± 6.13	17.35 ± 3.06	<0.05^a^^,^^b^
25(OH)D < 30 (ng/mL)	95	87.4%(83/95)	59	83.1%(49/59)	20/16	85%(17/20)	100%(16/16)	<0.05^a^^,^^b^
25(OH)D < 20 (ng/mL)	95	47.4%(45/95)	59	40.7%(24/59)	20/16	45%(9/20)	75%(12/16)	<0.05^a^^,^^b^
Total serum calcium (mmol/L)	95	2.42 ± 0.13	59	2.47 (2.38,2.54)	20/16	2.42 ± 0.86	2.36 ± 0.11	<0.05^a^
Serum phosphorus (mmol/L)	95	1.34 ± 0.32	59	1.53 ± 0.25	20/16	1.04 (0.97,1.13)	1.1 ± 0.12	<0.05^a^
Serum PTH (pg/mL)	94	33.98 (23.58,46.59)	58	32.13 (21.83,48.32)	20/16	39 ± 13.46	32.92 (30.73,47.45)	>0.05
Serum ALP (U/L)			58	239 (175,301)	19/16	96.89 ± 27.8	71.69 ± 19	<0.05^a^^,^^b^
Serum β-CTX (ng/L)			55	1,060 (818,1,492)	19/15	358.6 ± 180	272.1 ± 176	<0.05^a^
Serum OC (ng/mL)			56	91.89 (62.03,143.7)	19/14	25.18 ± 8.15	19.47 (17.56,29.68)	<0.05^a^

Data are shown as mean ± standard deviation or median (Q1, Q3).

*p* values represent the significance of the difference between two groups.

The confidence interval is 95%.

aBMD, areal bone mineral density; 25(OH)D, 25-hydroxyvitamin D; PTH, intact parathyroid hormone; ALP, alkaline phosphatase; β-CTX, beta cross-linked C-terminal telopeptide of type 1 collagen; OC, osteocalcin.

a Significantly different between ≤ 18 and > 18 groups.

b Significantly different between male and female in > 18 groups.

**Figure 2:**
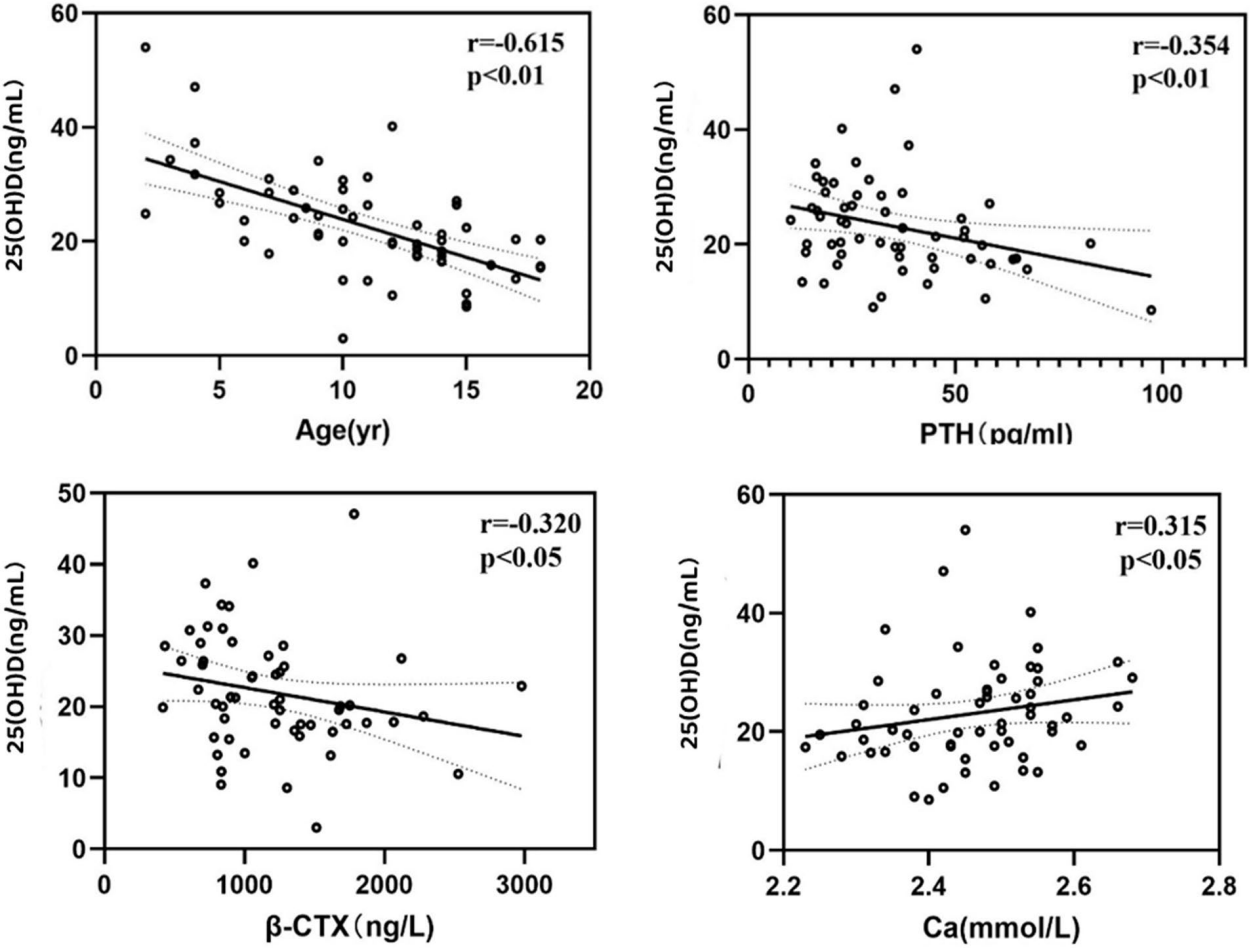
The correlation between 25OHD and other factors levels in under 18 group.

In the group under 18 years old, due to no statistical difference in means between genders and similar clinical and biochemical characteristics (*p* = 0.283), both girls and boys were analyzed together. The average serum 25(OH)D level was 22.71 ng/mL (±9.00), ranging from 3 to 54.03 ng/mL. A total of 49 patients (83.1%) had 25(OH)D concentrations below 30 ng/mL, 24 (40.7%) below 20 ng/mL, and 10 (16.9%) had sufficient levels. Serum 25(OH)D levels negatively correlated with age (r = −0.615, *p* < 0.01) and PTH (r = −0.354, *p* < 0.01), and positively with serum calcium (r = 0.315, *p* < 0.05), and negatively with β-CTX (r = −0.320, *p* < 0.05) (Figure 2). A negative correlation was observed between 25(OH)D levels and BMI (r = −0.237, *p* < 0.05). No correlation was found between serum 25(OH)D levels and height Z-score, weight Z-score, serum phosphorus, ALP, OC, BMD Z-score of femoral neck, trochanter, total hip or L1-L4 lumbar spine (Table 2).

**Table 2:** Correlates of vitamin D levels in OI patients.

Variables	Relevance/*p*			
	Total	≤18	>18-male	>18-female
Age at survey	r_s_ = −0.343/*p* < 0.01	r_s_ = −0.622/*p* < 0.01	r = 0.468/*p* < 0.05	r = 0.268/*p* = 0.315
Height (cm)	r_s_ = −0.276/*p* < 0.01	r_s_ = 0.139/*p* = 0.295	r = 0.091/*p* = 0.702	r = −0.211/*p* = 0.432
Height-Z	r = −0.281/*p* < 0.01	r_s_ = −0.011/*p* = 0.932	r = 0.227/*p* = 0.336	r = 0.094/*p* = 0.729
Weight (kg)	r = −0.151/*p* = 0.145	r_s_ = −0.299/*p* < 0.05	r = 0.163/*p* = 0.491	r = 0.285/*p* = 0.284
Weight-Z	r = −0.333/*p* < 0.01	r = −0.441/*p* < 0.01	r_s_ = −0.056/*p* = 0.863	r = −0.236/*p* = 0.484
BMI	r_s_ = −0.009/*p* = 0.939	r_s_ = −0.061/*p* = 0.669	r_s_ = 0.133/*p* = 0.681	r = −0.209/*p* = 0.537
aBMD L1-L4	r = −0.295/*p* < 0.01	r = −0.362/*p* < 0.01	r = 0.171/*p* = 0.47	r_s_ = −0.424/*p* = 0.102
aBMD L1-L4-Z	r_s_ = 0.066/*p* = 0.572	r_s_ = −0.008/*p* = 0.958	r = 0.291/*p* = 0.334	r_s_ = −0.238/*p* = 0.457
aBMD FN	r = −0.265/*p* < 0.05	r = −0.322/*p* < 0.05	r_s_ = 0.051/*p* = 0.83	r = −0.171/*p* = 0.528
aBMD FN-Z	r_s_ = 0.039/*p* = 0.738	r_s_ = 0.01/*p* = 0.996	r_s_ = 0.242/*p* = 0.426	r = −0.245/*p* = 0.443
aBMD total hip	r = 0.361/*p* < 0.01	r_s_ = 0.315/*p* < 0.05	r = 0.278/*p* = 0.234	r = 0.210/*p* = 0.435
aBMD total hip-Z	r = 0.246/*p* < 0.05	r = 0.234/*p* = 0.075	r = 0.05/*p* = 0.834	r = 0.16/*p* = 0.555
Ca	r_s_ = −0.343/*p* < 0.01	r_s_ = −0.622/*p* < 0.01	r = 0.468/*p* < 0.05	r = 0.268/*p* = 0.315
P	r_s_ = −0.276/*p* < 0.01	r_s_ = 0.139/*p* = 0.295	r = 0.091/*p* = 0.702	r = −0.211/*p* = 0.432
PTH	r_s_ = −0.327/*p* < 0.01	r_s_ = −0.354/*p* < 0.01	r = −0.159/*p* = 0.504	r_s_ = 0.141/*p* = 0.602
ALP		r_s_ = 0.183/*p* = 0.169	r = −0.286/*p* = 0.236	r = 0.284/*p* = 0.287
β-CTX		r_s_ = −0.320/*p* < 0.05	r = −0.132/*p* = 0.591	r_s_ = −0.004/*p* = 0.990
OC		r_s_ = −0.157/*p* = 0.251	r = −0.093/*p* = 0.705	r_s_ = 0.341/*p* = 0.233
OI Type	r_s_ = 0.14/*p* = 0.204	r_s_ = 0.242/*p* = 0.081	r_s_ = 0.038/*p* = 0.874	r_s_ = 0.15/*p* = 0.578
Genotype	r_s_ = 0.023/*p* = 0.856	r_s_ = 0.344/*p* = 0.034	r_s_ = −0.412/*p* = 0.089	r_s_ = 0.463/*p* = 0.111
Season	r_s_ = 0.109/*p* = 0.292	r_s_ = 0.049/*p* = 0.712	r_s_ = −0.303/*p* = 0.272	r_s_ = −0.555/*p* = 0.076

BMI, body mass index; Ca, calcium; P, phosphate; PTH, intact parathyroid hormone; ALP, alkaline phosphatase; β-CTX, beta cross-linked C-terminal telopeptide of type 1 collagen; OC, osteocalcin; OI, osteogenesis imperfecta; r, Pearson correlation coefficient; r_s_, Spearman correlation coefficient.

In the group aged over 18 years, due to statistical differences in mean values between male and female groups, the analysis was conducted separately for men and women. The average serum 25(OH)D level in males was 22 ± 6.13 ng/mL, and in females, it was 17.35 ± 3.06 ng/mL. 90% of adult OI patients had serum 25(OH)D concentrations below 30 ng/mL, which reached 100% in the female group. In male patients, a strong negative correlation was observed between the 25(OH)D level categories and the type of OI, the severity of OI phenotype (τb = −0.546, *p* < 0.05). Additionally, no correlation was found between serum 25(OH)D concentrations and other factors within both groups.

## Discussion

In this retrospective study, the patients were collected from the East China region (N 21°00 ~ 38°15). The children and adolescent patients had not received bisphosphonate treatment, and the adult patients had not received bisphosphonate treatment within the last 5 years, closely representing the original serum 25(OH)D levels of OI patients in this region.

Overall (n = 95, male 63/female 32), 83 patients (87.4%) had serum 25(OH)D concentrations below 30 ng/mL, 45 patients (47.4%) had levels below 20 ng/mL, and 3 patients (3.2%) had levels below 10 ng/mL. A large-scale study in Northern China on OI populations mentioned that 29.8% (104/349) and 47.3% (165/349) of OI patients were found to have vitamin D insufficiency (21–29 ng/mL) and deficiency (<20 ng/mL), respectively, and there was a negative correlation between 25(OH)D levels and the age of OI patients (29). Similar to this study, we also observed a negative correlation between 25(OH)D levels and the age of our patients, which could be due to children and adolescents being in the growth and development stage, making more effective use of vitamin D to support bone and overall development. Moreover, compared to adults and middle-aged individuals who might reduce outdoor activities due to work and other responsibilities, children and adolescents generally engage in more outdoor activities and have longer sun exposure. At the same time, analysis of indicators that do not show significant level differences due to age shows a negative correlation between serum 25(OH)D levels and PTH levels, which is also a secondary effect caused by changes in vitamin D levels. There is a positive correlation with serum calcium and phosphorus levels, reflecting the role of vitamin D in the absorption of calcium and phosphorus in the intestine within a certain range, regulating the balance of blood calcium and phosphorus. Dividing serum 25(OH)D levels into different concentration levels, the analysis shows a negative correlation between BMI and the level of 25(OH)D.

We observed that 83% of OI children and adolescents had insufficient or deficient levels of 25(OH)D (<30 ng/mL), with 47.4% of patients having serum 25(OH)D concentrations below 20 ng/mL. Similarly, other studies on OI patients and the general pediatric and adolescent population also showed a widespread deficiency in vitamin D levels. Edouard’s (25) study indicated that 69% of OI children and adolescents had insufficient or deficient 25(OH)D levels; a study in North America (19) found a 79.5% prevalence of vitamin D deficiency and insufficiency in OI children and adolescents; and a study in Brazil (20) classified 88.4% of OI children as vitamin D insufficient or deficient. In these studies, and in our own, a common finding was a negative correlation between serum 25(OH)D levels and age, similar to the age trend of vitamin D deficiency found in the general pediatric and adolescent population. In the Asian region, a study in South Korea (30) showed that 64.2% of adolescent females and 72.6% of adolescent males had 25(OH)D levels <20 ng/mL; a survey of vitamin D levels in Chinese children and adolescents found that those over 2 years old generally had low levels of vitamin D, with the highest deficiency rate of 46.4% occurring in adolescents aged 12–16 years (31).

Studies have shown that elder children and overweight children are more likely to be deficient in vitamin D (32). A survey of 25(OH)D levels in adolescents with obesity (33) (non-diabetic patients, aged 11–15) found that 40% of the subjects had 25(OH)D levels <20 ng/mL, and it was discovered that HOMA-IR (homeostasis model assessment for insulin resistance) is negatively correlated with vitamin D levels. A study in the United States involving high school students (aged 14–18) indicated a significant negative correlation between 25(OH)D levels and all obesity measurements, including BMI percentile, waist circumference, total fat mass, body fat percentage, visceral adipose tissue, and subcutaneous abdominal fat tissue (34). Research on adolescents (aged 15–19) from Northeast Brazil (35) found that 57.3% of the subjects had 25(OH)D levels <30 ng/mL, with adolescent females having a higher deficiency rate than males, primarily due to lower calcium intake and being overweight/obese. Our data analysis shows that the higher the BMI, the lower the serum 25(OH)D levels, indicating that, similar to the general pediatric and adolescent population, being overweight is a risk factor for vitamin D deficiency in OI children and adolescents.

In children with OI, the 25(OH)D levels are negatively correlated with parathyroid hormone concentrations and the bone resorption marker β-CTX, suggesting that low 25(OH)D content may have a detrimental effect on bone (36). Low levels of 25(OH)D may lead to insufficient calcium absorption, thereby stimulating PTH secretion to adapt to the higher bone formation rate associated with growth (37), increasing bone resorption, and thus releasing more calcium. No correlation between 25(OH)D and OC was observed, which may be due to the significant influence of factors such as vitamin K status and renal function on OC (38).

Different from Edouard’s previous retrospective study (25), in our patients, 73% of the samples with 25(OH)D levels were collected in autumn or winter, and no correlation was found between season and serum 25(OH)D levels. Previous studies (22, 23, 25) have shown that the type of OI is a significant independent predictor of 25(OH)D levels. Therefore, we included the type of OI, i.e., the severity of OI, as one of the influencing factors and predicted whether the type of COL1A1/COL1A2 mutation related to OI severity is also an influencing factor for 25(OH)D levels [OI severity is related to the degree and manner in which the type I collagen gene is affected: the first type is haploinsufficiency, including nonsense mutations, frameshift mutations, and splice mutations, leading to a reduction in the amount of type I collagen, often resulting in the milder Type I OI; the second type is caused by helix mutations due to glycine substitution in the Gly-X-Y triple helical domain, leading to abnormal type I collagen structure, often resulting in the more severe Types III and IV OI (39, 40)]. However, after analysis, no statistically significant differences were observed between different types of OI and gene mutation types, nor was there a correlation between them and 25(OH)D levels.

Insufficient serum 25(OH)D levels cause a drop in free calcium concentration, immediately recognized by the parathyroid glands. To maintain calcium homeostasis, PTH increases, leading to increased renal calcium retention and more calcium release through interaction with osteoblasts, thereby increasing bone turnover (41). However, the correlation between vitamin D levels and bone density has not been confirmed. Some studies have observed a positive correlation between 25(OH)D levels and bone density in healthy children (42–44), while many studies have not observed such a correlation in healthy subjects (45, 46). Many people do not seem to suffer bone loss or insufficient mineralization due to low 25(OH)D levels. Whether this is related to their diet (such as intake of calcium or of calcium binders like phytates) or other factors (such as the efficiency of renal calcium conservation) is not clear (47). In the OI population, there is rarely a precise correlation observed between 25(OH)D levels and bone density. Except for Zambrano et al., who reported a positive correlation between LS-BMD Z-scores and serum 25(OH)D levels in OI children (20), and Edouard et al., who reported that for every 1 nmol/L increase in serum 25(OH)D, there was an increase of 0.008 in LS-BMD Z-scores in children and adolescents with type I, III, and IV OI (25), this conclusion was refuted in another study by the same authors, which found no evidence that serum 25(OH)D levels between 13 and 103 nmol/L were related to differences in bone mineralization, metabolism, or bone mass in OI children (48). Our study also did not observe a correlation between 25(OH)D levels and bone density levels, as bone density is influenced by many factors such as age, gender, hormone levels, genetic factors, and other nutritional statuses, which may mask the effects of 25(OH)D on bone density.

It is noteworthy that in adult male patients, a negative correlation was observed between the levels of 25(OH)D and OI types, indicating lower 25(OH)D levels in males with more severe phenotypic manifestations of OI. This could be due to more significant skeletal health challenges requiring greater nutritional support, including vitamin D, which might not be sufficiently met in daily diet. Additionally, the fragility of bones and limitations in physical activity could lead to less outdoor activity, resulting in reduced synthesis of vitamin D due to less sun exposure. Obesity, which has been shown to correlate with lower serum 25(OH)D levels, might also develop due to limited activity. The differences between subgroups could be attributed to the fact that for children and adolescents, who are in their growth and development stages, bone metabolism and hormonal levels are significantly different from adults. As OI children grow older, especially upon reaching adulthood, hormonal levels (such as sex hormones) change, and symptoms and severity of bone defects in patients with more severe OI types worsen. Additionally, lifestyle and environmental factors at different ages might also impact vitamin D levels, such as differences in sun exposure, dietary habits, and physical activities. For adult females, the differences might arise from physiological disparities in bone health and metabolism between males and females. For example, differences in sex hormone levels might affect bone metabolism and the efficacy of vitamin D. These factors could account for the observed negative correlation between 25(OH)D levels and OI types across subgroups. These findings may indicate that the pathophysiology of OI varies among different genders and age groups, and the role of vitamin D may also differ across these groups. A deeper understanding of these findings and their potential clinical applications might require more extensive research, including larger sample sizes and more complex statistical analyses.

It is well-known that vitamin D is crucial for the normal development and protection of bones. Vitamin D deficiency affects calcium metabolism, osteoblast activity, matrix ossification, bone remodeling, and bone density (49). Generally, research suggests that vitamin D impacts bone metabolism as follows: Vitamin D enhances intestinal calcium absorption and maintains serum calcium and phosphate levels, facilitating normal bone mineralization (47). Additionally, vitamin D acts on osteoblasts through the VDR, promotes osteoclast formation, stimulates bone resorption (50), and enhances bone growth and remodeling (51). Studies have suggested that vitamin D should be supplemented before starting BPs treatment to ensure adequate serum vitamin D levels (52). Ensuring sufficient levels of 25(OH)D is an important component of clinical management for osteogenesis imperfecta, which helps improve bone health, reduce fracture risk, and enhance overall treatment outcomes for patients. It can be recommended that all studies on OI patients should be conducted after achieving sufficient vitamin D serum levels to optimize patient management and reduce confounding factors. Research shows that an individual’s initial baseline level of 25(OH)D prior to vitamin D supplementation can affect their response to supplementation (53). An RCT study showed that the impact of baseline 25(OH)D levels on Alendronate treatment alone indicated that patients with vitamin D deficiency had poorer response to the reduction of bone resorption marker CTX, while patients with sufficient baseline 25(OH)D levels had better response; Moreover, supplementing with vitamin D can not only help control PTH levels, but also enhance the reduction effect of bone resorption markers, especially in patients with high PTH levels or vitamin D deficiency (54). So studying baseline 25(OH)D levels in before medication is of research value, especially for OI patients who are prone to fractures. A large sample birth cohort study from Germany did not find a correlation between 25(OH)D and OC but reported a negative correlation between 25(OH)D and β-CTX (14). This is similar to our study findings. Some RCTs indicate that vitamin D supplementation affects bone metabolic markers (55, 56). However, several studies did not exhibit significant effects of vitamin D supplementation on bone metabolic markers (57, 58). Overall, the information is relatively limited and contradictory. Bone metabolic markers, as indicators of fracture or osteoporosis risk, are influenced by various systemic and local factors, as well as environmental and dietary factors (59). Our study, possibly due to factors like sample size, may show some false negatives but still reflects the situation of the OI population in East China to some extent. Our study essentially eliminates the interference of bisphosphonates on bone density and vitamin D levels, providing baseline 25(OH)D data for a large OI population across multiple age groups in East China, analyzing and summarizing the correlation between vitamin D levels and bone metabolic markers, bone density, and other factors, thus enriching a part of the research landscape on vitamin D in OI patients. This retrospective study also has certain limitations, as a retrospective study, we have many confounding factors that cannot be fully considered due to incomplete case collection, and due to the inclusion criteria, the sample size is not large enough, especially for adult OI patients. In future research, we plan to use standardized questionnaires to collect patient lifestyle information, including detailed dietary logs and physical activity records, such as recording the daily consumption of milk and carbonated drinks, outdoor exercise time, sunscreen use, etc. (19, 20, 23), and use multivariate analysis methods to control for these lifestyle factors, in order to help us better understand the independent effects and potential interactions of these lifestyle factors on vitamin D levels in OI patients.

## Conclusion

This study on osteogenesis imperfecta (OI) in East China reveals significant vitamin D deficiency and insufficiency in pediatric, adolescent and adult OI patients, similar to global trends. The findings underscore the inverse correlation between serum 25(OH)D levels and factors like age, BMI, and parathyroid hormone concentrations. No seasonal changes in 25(OH)D levels and no correlation with bone density parameters were observed. Interestingly, there is a correlation between 25(OH)D levels and the severity of OI in adult male OI patients, potentially due to physiological and hormonal differences. These insights are crucial for understanding the vitamin D status of OI patients across different age groups, contributing to better clinical management of OI patients, highlighting the importance of regular monitoring and personalized vitamin D supplementation in OI patients to improve bone health outcomes. Future research should focus on understanding the underlying mechanisms and long-term effects of vitamin D management in different demographic groups, particularly in adult males where severity correlations were observed.

## Data availability statement

The original contributions presented in the study are included in the article/supplementary material, further inquiries can be directed to the corresponding authors.

## Ethics statement

The studies involving humans were approved by the Ethics Committee of Shanghai Jiao Tong University School of Medicine Affiliated Sixth People’s Hospital. The studies were conducted in accordance with the local legislation and institutional requirements. Written informed consent for participation in this study was provided by the participants’ legal guardians/next of kin.

## Author contributions

YJ: Conceptualization, Investigation, Methodology, Writing – original draft, Writing – review & editing. YM: Formal analysis, Methodology, Writing – review & editing. YT: Data curation, Software, Writing – review & editing. LS: Data curation, Software, Writing – review & editing. SX: Investigation, Supervision, Writing – review & editing. HZ: Formal analysis, Funding acquisition, Resources, Supervision, Writing – review & editing. ZZ: Formal analysis, Funding acquisition, Resources, Supervision, Writing – review & editing.
